# A critical analysis of Powell’s results on the interdivision time distribution

**DOI:** 10.1038/s41598-019-44606-4

**Published:** 2019-06-03

**Authors:** Vincent Quedeville, Jérôme Morchain, Philippe Villedieu, Rodney O. Fox

**Affiliations:** 1 FERMAT, Université de Toulouse, CNRS, INPT, INSA, UPS, Toulouse, France; 2 Laboratoire d’Ingénierie des Systèmes biologiques et des Procédés, INSA de Toulouse, 135 Avenue de Rangueil, 31400 Toulouse, France; 3 Institut de Mathématiques de Toulouse, Université de Toulouse, F-31062 Toulouse, France; 4 ONERA/DMPE, Université de Toulouse, F-31055 Toulouse, France; 5 Department of Chemical and Biological Engineering, Iowa State University, 618 Bissell Road, Ames, Iowa 50011-1098 USA

**Keywords:** Applied mathematics, Computational models

## Abstract

The cell-age and interdivision-time probability density functions (PDFs) have been extensively investigated since the 1940s due to their fundamental role in cell growth. The pioneering work of Powell established the first relationship between the interdivision-time and cell-age PDFs. In the literature, two definitions for the interdivision-time PDF have been proposed. One stands for the age-at-rupture PDF and is experimentally observable, whereas the other is the probability density that a cell divides at a certain age and is unobservable. From Powell’s results pertaining to the unobservable interdivision-time PDF, Painter and Marr derived an inequality that is true but is incorrectly used by experimentalists to analyse single-cell data. Unfortunately, the confusion between these two PDFs persists. To dissipate this confusion, exact relationships between the cell-age and the interdivision-time PDFs are derived in this work from an age-structured model, which can be used by experimentalists to analyse cell growth in batch and continuous culture modes.

## Introduction

Understanding biological population dynamics in a fermenter has been of crucial importance in bio-process engineering and many other fields, such as pharmacology, that require a mass production of metabolic by-products. A strain will grow differently in a batch or continuous fermenter, to the extent that one population will exhibit different characteristics depending on the culture conditions and the observed features cannot be compared. In particular, in an open system, the fermenter dilution rate, *D*, will determine the ensemble-averaged behaviour, such as the mean age or mean interdivision time, in other words the cell-cycle duration. As early as 1956 Powell^1^ hinted at the seminal conclusion that a continuous culture’s observed mean interdivision time, $\langle \tau \rangle$, must be less that the so-called population doubling time, meaning that as soon as the interdivision-time distribution is asymmetric, the healthier cells will contribute more to maintaining a steady-state cell number than their less active counterparts. To date, that article has been cited in 372 research works, with significant interest from mathematicians^2^, physicists^3^, chemists^4^ and biologists^5^ on a variety of perspectives pertaining to the cell-cycle dynamics and the marginal distributions in different observable properties such as age, size or cell content. In the last decade, the development of microfluidic devices has broadened the biologists’ horizons and given more accurate statistical information regarding the cell-cycle processes^6–9^, allowing modelling assumptions to be tested against experimental results. However, Powell’s logical reasoning leading to $\langle \tau \rangle \le \,\mathrm{ln}\,2$/*D* is not a consensus view in the mathematical modelling community; indeed, in 1967 Painter & Marr^10^ demonstrated the exact opposite inequality starting from Powell’s work and no one has, to the authors’ knowledge, questioned this assessment to date. If anything, Painter & Marr’s demonstration has paved the way for experimental and analytical work, i.e.^9,11,12^, attempting to consolidate Painter & Marr’s viewpoint.

The very notion of interdivision-time distribution can embrace different quantities in spite of a common definition of the concept (i.e. the time elapsed between two consecutive division events of an observed organism), and no consensus has been reached to date on the relationship between these quantities. Consequently, some semantics are required to provide a framework for the analytical results presented in this work and for their comparison with experimental data.

- A cell’s age is defined as the time elapsed since the division event that produced it (that is the age in the cycle), entailing that the quantity is reset to zero after each recorded rupture. It does not encompass the lineage’s longevity that will be called the “age in the system”. The latter is tantamount to the abiotic phase’s lifespan because this time interval is just the residence time in the fermenter.
- An observable interdivision-time distribution, *g*, stands for a collection of recorded cell-cycle durations for which labels such as “generation time”^1^ or “doubling time”^7^ exist in the literature. The vocabulary is here borrowed from^6^ so that interdivision time will be synonymous with the cell’s age at rupture. As a consequence, *g* refers to Powell’s so-called “carrier distribution” and $g(a)da$ defines the conditional probability that a cell that has divided has done so between age *a* and $a+da$. It is essential to notice that *g* captures the Markovian nature of the cell cycle process and reports the fact that a cell has reached age *a* in the system. Throughout the article, $\int \,ag(a)da$ will be called ${\tau }_{obs}$.
- The unobservable interdivision-time distribution *h*, used by Powell^1^, is such that $h(a)da$ is the probability that a newly formed organism will have a generation time in the range [*a*, $a+da$]. Its first moment $\int \,ah(a)da$ will be called ${\tau }_{uno}$.

Powell sheds light on the dissimilitudes between these two interdivision-time distributions. This distinction is relevant for both batch and continuous conditions for different reasons. In an open fermenter, a cell’s biological development must be considered along with its residence time so an organism’s interdivision time could refer to an unobservable and, hence, unmeasurable event (for instance from statistical considerations, from any steady-state group of tracked particles, half of them will be washed out before dividing). In batch culture, the younger elements outnumber their ancestors due to the biotic phase’s exponential growth and the statistical extra weight conferred on the less probable quicker interdivision times over a much larger share of the population pushes the age-at-rupture distribution to the left. Experimentally, the available data regarding interdivision time pertain to the age at rupture, and the PDF’s first moment is well approximated by the data set’s arithmetic mean, provided the collected data set is large enough. In 1956, Powell^1^ claimed he did produce an experimental equivalent for *h* and fitted the histogram with a Pearson type-III distribution. However, he remarked in 1964^13^ that “the generation times of the organisms which have, at a given time, completed their life span during the previous history of the culture do not compose” *h*; “they compose the carrier distribution” *g*. In fact, only information regarding the cell’s age at rupture is available to experimentalists and, hence, it cannot be interpreted with analytical results intended for the unobservable interdivision-time PDF *h*. In 1967, Painter & Marr^10^ extracted a lower bound on the first moment of Powell’s interdivision-time distribution ${\tau }_{uno}$ and confused it with the observable mean interdivision time ${\tau }_{obs}$, prompting some equivocal assertions (a very good recent example being^9^) by lack of consensus. Keeping these considerations in mind, this work aims to reconcile the persistent misunderstanding about these distinct paradigms, and on presenting exact analytical results regarding the observable interdivision-time distribution that are accessible to experimentalists. To illustrate the most important points, numerical examples are provided from Monte–Carlo simulations of a population balance model. Furthermore, unlike^14^ or^8^ where the so-called “timer” or “adder” models are given prominence, by virtue of^15^ the rupture process will be assumed to be a function of the cell length.

The first part of the paper presents the general framework of population balance modelling in the context of microbial populations, the particular population balance equation (PBE) chosen for the present study and the mathematical definition of the age and observable interdivision-time distributions. The second part is devoted to analytical results leading to relationships valid for the age and interdivision-time PDFs that are observable from batch and chemostat experimental measurements. These analytical results are further underpinned by numerical simulations using a Monte–Carlo algorithm. In the discussion, the results from the previous section are compared to experimental data from the literature. A resolution of the seemingly contradictory conclusions in Powell’s and Painter and Marr’s works is provided.

## Mathematical Background and Definitions

### General formulation of a PBE for biological populations

Beginning with work in the 1960s^16,17^, PBEs have provided a general framework to describe the biological response to a user-defined experimental set-up. In this context, an inner coordinate is understood as a marginal variable and its law is retrieved through integration with respect to all other dimensions. When biological modelling of a continuous fermenter is addressed, the age-structured PBE takes the form

1 $$\begin{array}{l}\frac{\partial }{\partial t}n(t,a,{\boldsymbol{\xi }})+\frac{\partial }{\partial a}n(t,a,{\boldsymbol{\xi }})+{\boldsymbol{\nabla }}\cdot [\dot{{\boldsymbol{\xi }}}n(t,a,{\boldsymbol{\xi }})]\\ \begin{array}{rcl}\,+\,\gamma (a,{\boldsymbol{\xi }})n(t,a,{\boldsymbol{\xi }})+D\,n(t,a,{\boldsymbol{\xi }}) & = & 0\\ \,n(t,0,{\boldsymbol{\xi }}) & = & 2\,{\int }_{{{\rm{\Omega }}}_{\xi }}\,{\int }_{0}^{\infty }\,\,\gamma (a^{\prime} ,{\boldsymbol{\xi }}^{\prime} )\\  &  & \times \,K({\boldsymbol{\xi }},{\boldsymbol{\xi }}^{\prime} ,a^{\prime} )n(t,a^{\prime} ,{\boldsymbol{\xi }}^{\prime} )da^{\prime} d{\boldsymbol{\xi }}^{\prime} \\ \,\dot{{\boldsymbol{\xi }}}n(t,a,{\boldsymbol{\xi }}){|}_{{\boldsymbol{\xi }}\in \partial {{\rm{\Omega }}}_{\xi }} & = & 0,a\in [0,+\,\infty [\end{array}\end{array}$$

with ${\boldsymbol{\xi }}\in {{\rm{\Omega }}}_{\xi }\subset {{\mathbb{R}}}^{n}$, $n\ge 1$, the vector of inner coordinates, $\dot{{\boldsymbol{\xi }}}$ their rate of change, *K* the redistribution kernel, and $n(t,{\boldsymbol{\xi }})d{\boldsymbol{\xi }}$ the cell number in an infinitesimal domain of Lebesgue measure *d*${\boldsymbol{\xi }}$. In (1) *γ* (time unit^−1^) is the rupture function, or simply the cell-division frequency, and *D* (time unit^−1^) stands for the so-called dilution rate that drives both the input feed and cell washout to maintain the medium volume. In general, $\dot{{\boldsymbol{\xi }}}$ is a function of both ${\boldsymbol{\xi }}$ and the organisms’ environment, but its formulation has no impact on the section’s results. Hereinafter, *D* will be assumed constant and washout is assumed uniform with respect to any inner coordinate (i.e., the fermenter is perfectly mixed).

In a continuous fermenter, an equilibrium will be reached when the time derivative in (1) vanishes and this condition is referred to as steady state. In a batch fermenter, the absence of a washout term will allow the cell number to grow at will as soon as the initial conditions have faded away. This equilibrium is thoroughly discussed in^17^ and will be referred to as self-similar exponential growth. The time derivative in (1) does not vanish in this case, and the stability property will relate to the marginal distribution’s geometrical shape. In other words, the scaled quantity

$$\frac{n(t,a,{\boldsymbol{\xi }})}{{\int }_{{{\rm{\Omega }}}_{\xi }}\,n(t,a,{\boldsymbol{\xi }})d{\boldsymbol{\xi }}}$$

will be constant (or self similar) for any ${\boldsymbol{\xi }}\in {{\rm{\Omega }}}_{\xi }$.

### Application to *E*. *coli* population dynamics

Without loss of generality, this section will consecrate a two-dimensional PBE, ${\boldsymbol{\xi }}$ standing for the cells’ length $l\in [0,\bar{l}[$ (m), where $\bar{l}$ is the maximum possible cell length before division. In this section, no laws for $\dot{l}$ are yet required. Nonetheless, it is understood that such a process must be a decreasing function of *l* since it involves the internal transport of membrane proteins from the cytoplasm, which takes longer as the cell grows larger. Indeed, as noted by Nobs & Maerkl^7^, synthesis of cell-membrane components could be one such factor setting limits on the cell-doubling time, what seems universal enough to feature in any biological population modelling. Other patterns are conceivable though (as mentioned in^9^) but the lack of experimental data makes any consensus unattainable.

Also, the redistribution kernel obeys ${\int }_{0}^{l^{\prime} }\,P(l,l^{\prime} ,a^{\prime} )dl=1$, which is the mathematical counterpart of the biological hypothesis that a given cell gives birth to only two daughter cells during the division process.

Hence, (1) reads in this case:

2 $$\begin{array}{l}\frac{\partial }{\partial t}n(t,l,a)+\frac{\partial }{\partial l}[\dot{l}n(t,l,a)]+\frac{\partial }{\partial a}n(t,l,a)\\ \begin{array}{rcl}\,+\,\gamma (l,a)n(t,l,a)+D\,n(t,l,a) & = & 0\\ \,n(t,l,0) & = & 2\int \,{\int }_{l^{\prime}  > l}\,\gamma (l^{\prime} ,a^{\prime} )\\  &  & \times \,P(l,l^{\prime} ,a^{\prime} )n(t,l^{\prime} ,a^{\prime} )dl^{\prime} da^{\prime} \\ \,\dot{l}n(t,l,a){|}_{l=0} & = & 0=\dot{l}n(t,l,a){|}_{l=\bar{l}}\end{array}\end{array}$$

In the system (2), a null-flux condition is assumed at the domain boundary in length, what is tantamount to the claim that no cell can grow beyond a certain length that challenges its biomechanical structure. From physical grounds, it will similarly be assumed that no cells will reach infinite age.

### Definition of PDFs

Considering that $n(t,l,a)$ refers to the number density of cells with length *l*, age *a* at time *t* in a continuous reactor and *γ* is the cell-division frequency, the function

3 $$\begin{array}{c}g:{{\mathbb{R}}}_{+}\to {{\mathbb{R}}}_{+}\\ a\mapsto g(a)=\frac{\int \,\gamma (l,a)n(t,l,a)dl}{\iint \,\gamma (l,a)n(t,l,a)dlda}\end{array}$$

will designate the interdivision-time PDF as it is observed in experimental measurements. Its moments are denoted by $\langle {\tau }^{k}\rangle =\int \,{a}^{k}g\,da$, $k\ge 1$, $\langle {\tau }^{1}\rangle$ coinciding with ${\tau }_{obs}$. It is brought to the reader’s attention that $g(a)da$ is not the probability that a cell divides between age *a* and $a+da$. Instead, *g* denotes what Powell called the carrier distribution ${{\mathscr{C}}}_{D}$ in his 1956 article^1^ and corresponds to the observed cell-cycle duration.

Furthermore, the cell-age PDF *f* can be retrieved by integrating (2) with respect to *l*:

4 $$f(t,a)=\frac{\int \,n(t,l,a)dl}{\iint \,n(t,l,a)dlda}=\frac{N(t,a)}{N(t)}$$

where $N(t,a)=\int \,n(t,l,a)dl$ and $N(t)=\iint \,n(t,l,a)dlda$. Thus $f(a)da$ is the probability that a cell in the reactor has an age between *a* and $a+da$, and *f* is therefore tantamount to Powell’s $\varphi$ in his 1956 article^1^.

Comparing (3) to (4), we observe that the interdivision-time PDF is weighted by the cell-division frequency, while the cell-age PDF is not. At steady state, or under self-similar conditions, both PDFs will be independent of *t*.

## Analytical and Numerical Results

In this section, we establish exact results concerning the interdivision-time and cell-age PDFs arising from the solution to the PBE introduced above.

### Steady-state relation between *f* and *g* in a continuous fermenter

From the definition of *f* provided in (4), one gets:

5 $$\frac{\partial }{\partial t}f(t,a)=\frac{1}{N(t)}\frac{\partial }{\partial t}N(t,a)-\frac{f(t,a)}{N(t)}\frac{\partial }{\partial t}N(t)$$

The first term on the right-hand side of (5) is obtained through an integration of (2) with respect to *l*, i.e.

6 $$\begin{array}{l}\int \,\frac{\partial }{\partial t}n(t,l,a)dl+\int \,\frac{\partial }{\partial l}[\dot{l}n(t,l,a)]\,dl+\int \,\frac{\partial }{\partial a}n(t,l,a)dl\\ \,+\,\int \,\gamma (l,a)n(t,l,a)dl+D\,\int \,n(t,l,a)dl=0\end{array}$$

In (6), the first term on the left-hand side designates $N(t,a)$’s time derivative and the null-flux boundary condition forces the second term to vanish. Furthermore,

$$\int \,\frac{\partial }{\partial a}n(t,l,a)dl=\frac{\partial }{\partial a}N(t,a)\,{\rm{and}}\,D\,\int \,n(t,l,a)dl=D\,N(t,a)$$

Hence,

7 $$\frac{\partial }{\partial t}N(t,a)+\frac{\partial }{\partial a}N(t,a)+\int \,\gamma (l,a)n(t,l,a)dl+D\,N(t,a)=0$$

The second term on the right-hand side of (5) is retrieved from the double integral of (2):

$$\begin{array}{l}\iint \,\frac{\partial }{\partial t}n(t,l,a)dlda+\iint \,\frac{\partial }{\partial l}[\dot{l}n(t,l,a)]\,dlda+\iint \,\frac{\partial }{\partial a}n(t,l,a)dlda\\ \,+\,\iint \,\gamma (l,a)n(t,l,a)dlda+D\,\iint \,n(t,l,a)dlda=0\end{array}$$

The same reasoning as before entails the conclusion that the first term on the left-hand side is in fact *N*(*t*)’s time derivative and the second term is null. Use of Fubini’s theorem and the fact that there is no cell with an infinite age in the system turns the third term into

8 $$\begin{array}{rcl}\int \,\int \,\frac{\partial }{\partial a}n(t,l,a)dadl & = & \int \,{[n(t,l,a)]}_{a=0}^{\infty }dl\\  & = & -\,2\,\int \,\iint \,\gamma (l^{\prime} ,a^{\prime} )P(l,l^{\prime} ,a^{\prime} )n(t,l^{\prime} ,a^{\prime} )dl^{\prime} da^{\prime} dl\\  & = & -\,2\,\iint \,\gamma (l,a)n(t,l,a)dlda\end{array}$$

Consequently,

9 $$\frac{\partial }{\partial t}N(t)-\iint \,\gamma (l,a)n(t,l,a)dlda+D\,N(t)=0$$

Combining (9) and (7) in (5) and referring to the definition of *f* in (4) yields an equation for the time evolution of *f*:

10 $$\begin{array}{rcl}\frac{\partial }{\partial t}f(t,a) & = & -\frac{\partial }{\partial a}f(t,a)-\frac{\int \,\gamma (l,a)n(t,l,a)dl}{N(t)}\\  &  & -\,\frac{f(t,a)}{N(t)}\,\iint \,\gamma (l,a)n(t,l,a)dlda\end{array}$$

A steady-state relationship between *f* and *g* can be derived from this equation. Indeed, at steady state, *f*’s derivative with respect to time vanishes (removing the time dependence) and it also follows from (9) that

11 $$\iint \,\gamma (l,a)n(l,a)dlda=DN$$

Therefore, from (10)

12 $$\begin{array}{rcl}\frac{df}{da}(a) & = & -\frac{\int \,\gamma (l,a)n(l,a)dl}{N}-Df(a)\\  & = & -\frac{\int \,\gamma (l,a)n(l,a)dl}{\int \,\gamma (l,a)}n(l,a)dlda\\  &  & \times \,\frac{\iint \,\gamma (l,a)n(l,a)dlda}{N}-Df(a)\end{array}$$

The last step consists in using (3), the definition of *g*, and (11) in (12) to get the desired relationship:

13 $$f^{\prime} (a)=-\,Dg(a)-Df(a)$$

### An analytical solution for the cell-age distribution at steady state in a continuous fermenter

The differential equation (13) can be solved using Duhamel’s formula and yields

14 $$f(a)=f(0){e}^{-Da}-D{e}^{-Da}\,{\int }_{0}^{a}\,{e}^{Da^{\prime} }g(a^{\prime} )da^{\prime} $$

One only needs to provide *f*(0) to completely define *f*. From the definition of *f*, given in (4), the null-age relation provided in (2) and the steady-state relation (11), the boundary condition reads:

15 $$\begin{array}{rcl}f(0) & = & \frac{\int \,n(l,0)dl}{\iint \,n(l,a)dlda}\\  & = & \frac{2\int \,\iint \,\gamma (l^{\prime} ,a^{\prime} )P(l,l^{\prime} ,a^{\prime} )n(l^{\prime} ,a^{\prime} )dl^{\prime} da^{\prime} dl}{N}\\  & = & \frac{2DN}{N}\\  & = & 2D\end{array}$$

Consequently the cell-age distribution at steady state reads:

16 $$f(a)=2D{e}^{-Da}-D{e}^{-Da}\,{\int }_{0}^{a}\,{e}^{Da^{\prime} }g(a^{\prime} )da^{\prime} $$

This result extends Ramkrishna’s^18^ work dealing with analytical and numerical solutions of age and size PBMs in a closed bioreactor. It is worth mentioning that (15) and (16) echo Powell’s equation (9)^1^. Both derivations complement each other since the cell-age PDF definitions are in fact identical (Powell’s $\varphi$ is equivalent to our *f*). However, Powell’s results involve an interdivision-time distribution that is unobservable from experiments contrary to our *g*.

### For a continuous fermenter at steady state *τ*_*obs*_ ≤ ln(2)/*D*

This result is obtained from rearranging (16) and taking the limit $a\to \infty$. One can first check that the application $a:\,\mapsto \exp (Da)f(a)$ is strictly decreasing on ${{\mathbb{R}}}_{+}$. Indeed:

$$\begin{array}{rcl}\frac{d}{da}(\exp (Da)f(a)) & = & D\,\exp (Da)f(a)\\  &  & +\,\exp (Da)\,(\,-\,Df(a)-Dg(a))\\  & = & -D\,\exp (Da)g(a) < 0\end{array}$$

since *g* is strictly positive on ${{\mathbb{R}}}_{+}$. As 0 is an obvious lower bound to $a\mapsto \exp (Da)f(a)$, the latter converges to a finite limit $\lambda \ge 0$. As a consequence:

$${\int }_{0}^{a}\,{e}^{Da^{\prime} }g(a^{\prime} )da^{\prime} =2-\frac{1}{D}{e}^{Da}f(a)\Rightarrow \mathop{\mathrm{lim}}\limits_{a\to \infty }\,{\int }_{0}^{a}\,{e}^{Da^{\prime} }g(a^{\prime} )da^{\prime} =2-\frac{\lambda }{D}$$

Then, developing the exponential into a power series and making use of Jensen’s inequality leads to:

$$\sum _{k\ge 0}\frac{{D}^{k}}{k!}\,{\int }_{0}^{\infty }\,a{^{\prime} }^{k}g(a^{\prime} )da^{\prime} =\sum _{k\ge 0}\,\frac{{D}^{k}}{k!}\langle {\tau }^{k}\rangle =2-\frac{\lambda }{D}\ge \sum _{k\ge 0}\,\frac{{D}^{k}}{k!}{\tau }_{obs}^{k}$$

and thus to

17 $$2-\frac{\lambda }{D}\ge {e}^{D{\tau }_{obs}}\iff {\tau }_{obs}\le \frac{\mathrm{ln}(2-\frac{\lambda }{D})}{D}\le \frac{\mathrm{ln}(2)}{D}$$

The last inequality does not prevent ${\tau }_{obs}$ from being equal to ln(2)/*D*, which would happen if all moments $\langle {\tau }^{k}\rangle$ were equal to ${\tau }_{obs}^{k}$. This would basically force *g* to be a Dirac delta function: ${\delta }_{a-\mathrm{ln}(2)/D}$. In this case though, the observable and unobservable distributions are identical and mirror the behaviour of an unstructured model. A preliminary conclusion was first formulated by Tyson & Hannsgen^2^, but the authors missed Powell’s^13^ remark pertaining to the difference between the two interdivision-time distributions, preventing their result from being applicable to actual experimental data.

**Figure 1:**
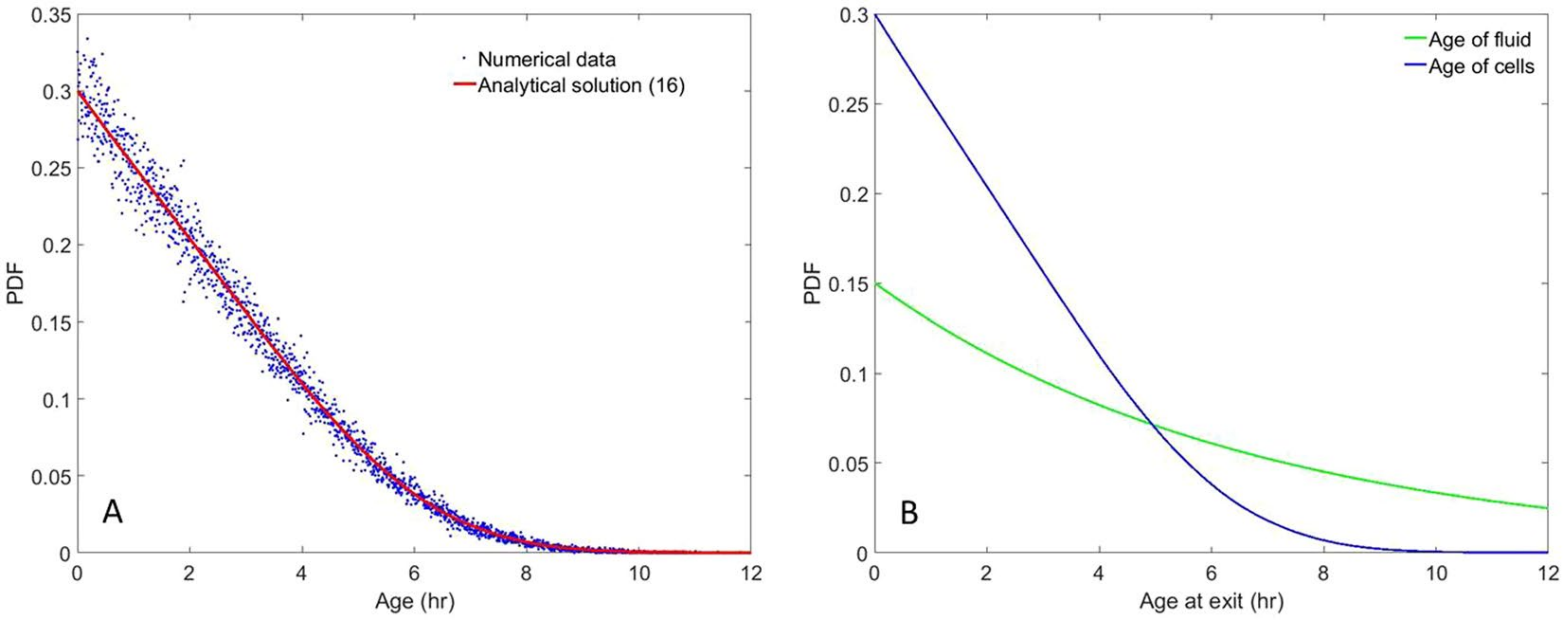
Steady-state results for continuous culture with $D=0.15\,{{\rm{hr}}}^{-1}$. (**A**) Cell-age PDF *f* from the Monte–Carlo simulation (blue points), compared with the analytical solution (red line) (16) where $\langle a\rangle \approx 2.360\,{\rm{hr}}$. (**B**) Cell-age PDF (blue line) compared with the fluid residence-time PDF (green line).

An additional conclusion that stems from (14) is that the outlet-age profile (that must be tantamount to the fermenter’s because of the uniform washout assumption) differs significantly from the liquid phase’s (i.e., *De*^−*Da*^), because the biological phase renewal is a consequence of two competing phenomena: dilution and cell division. A graphic comparison between the two residence time distributions is shown in Fig. 1.

### For a continuous fermenter at steady state 〈*a*〉 + *τ*_*obs*_ = 1/*D*

Taking the first moment of (16) yields

$$\begin{array}{rcl}\langle a\rangle ={\int }_{0}^{\infty }\,af(a)da & = & 2D\,{\int }_{0}^{\infty }\,a{e}^{-Da}da-D\,{\int }_{0}^{\infty }\,a{e}^{-Da}\,{\int }_{0}^{a}\,{e}^{Da^{\prime} }g(a^{\prime} )da^{\prime} da\\  & = & 2\,{\int }_{0}^{\infty }\,{e}^{-Da}da-D\,{\int }_{0}^{\infty }\,{e}^{Da^{\prime} }g(a^{\prime} )\,{\int }_{a^{\prime} }^{\infty }\,a{e}^{-Da}dada^{\prime} \\  & = & \frac{2}{D}-{\int }_{0}^{\infty }\,{e}^{Da^{\prime} }g(a^{\prime} )({\,-\,a{e}^{-Da}|}_{a^{\prime} }^{\infty }+{\int }_{a^{\prime} }^{\infty }\,{e}^{-Da}da)da^{\prime} \\  & = & \frac{2}{D}-{\int }_{0}^{\infty }\,ag(a)da-{\int }_{0}^{\infty }\frac{1}{D}{e}^{-Da}{e}^{Da}g(a)da\\  & = & \frac{2}{D}-{\tau }_{obs}-\frac{1}{D}\end{array}$$

and thus,

18 $$\langle a\rangle +{\tau }_{obs}=\frac{1}{D}$$

Using (17), an upper bound to ${\tau }_{obs}$ is obtained:

19 $${\tau }_{obs}\le \frac{\langle a\rangle \,\mathrm{ln}(2)}{1-\,\mathrm{ln}(2)}\approx 2.259\langle a\rangle $$

The same reasoning yields a relation between the second-order moments of *f* and *g*:

$$\begin{array}{rcl}\langle {a}^{2}\rangle  & = & {\int }_{0}^{\infty }\,{a}^{2}f(a)da\\  & = & 2D\,{\int }_{0}^{\infty }\,{a}^{2}{e}^{-Da}da-D\,{\int }_{0}^{\infty }\,{a}^{2}{e}^{-Da}\,{\int }_{0}^{a}\,{e}^{Da^{\prime} }g(a^{\prime} )da^{\prime} da\\  & = & {-2{a}^{2}{e}^{-Da}|}_{0}^{\infty }+2\,{\int }_{0}^{\infty }\,2a{e}^{-Da}da-{\int }_{0}^{\infty }\,{e}^{Da^{\prime} }g(a^{\prime} )\,{\int }_{a^{\prime} }^{\infty }\,D{a}^{2}{e}^{-Da}dada^{\prime} \\  & = & \frac{4}{{D}^{2}}-{\int }_{0}^{\infty }\,{e}^{Da^{\prime} }g(a^{\prime} ){-\,{a}^{2}{e}^{-Da}|}_{a^{\prime} }^{\infty }da^{\prime} \\  &  & -\,{\int }_{0}^{\infty }\,{e}^{Da^{\prime} }g(a^{\prime} )\,{\int }_{a^{\prime} }^{\infty }\,2a{e}^{-Da}dada^{\prime} \\  & = & \frac{4}{{D}^{2}}-\langle {\tau }^{2}\rangle -{\int }_{0}^{\infty }\,{e}^{Da^{\prime} }g(a^{\prime} ){-\,\frac{2}{D}a{e}^{-Da}|}_{a^{\prime} }^{\infty }da^{\prime} \\  &  & -\,{\int }_{0}^{\infty }\,{e}^{Da^{\prime} }g(a^{\prime} )\,{\int }_{a^{\prime} }^{\infty }\,\frac{2}{D}{e}^{-Da}dada^{\prime} \\  & = & \frac{4}{{D}^{2}}-\langle {\tau }^{2}\rangle -\frac{2}{D}{\tau }_{obs}-\frac{2}{{D}^{2}}\\  & = & \frac{2}{{D}^{2}}-\langle {\tau }^{2}\rangle -\frac{2}{D}{\tau }_{obs}\end{array}$$

yielding:

20 $$\langle {a}^{2}\rangle =\frac{2}{D}\langle a\rangle -\langle {\tau }_{obs}^{2}\rangle $$

Consider now an age-synchronised population, i.e., no variance in age is observed ($\langle {a}^{2}\rangle ={\langle a\rangle }^{2}$). Then using (18) and (20) one can determine whether a non-zero variance can exist in the interdivision-time distribution.

$$\langle {\tau }^{2}\rangle -{\tau }_{obs}^{2}=\frac{2}{D}\langle a\rangle -\langle {a}^{2}\rangle -\frac{1}{{D}^{2}}-{\langle a\rangle }^{2}+\frac{2}{D}\langle a\rangle =-\,2{\langle a\rangle }^{2}+\frac{4}{D}\langle a\rangle -\frac{1}{{D}^{2}}$$

The second-order polynomial would vanish for $D\langle a\rangle \in \{1-\sqrt{2}/2,1+\sqrt{2}/2\}$, the latter value being impossible given that $1+\sqrt{2}$/$2 > 1$. However, if $\langle a\rangle$ were equal to $(1-\sqrt{2}/2)D$, the mean interdivision time ${\tau }_{obs}$ would be $\frac{\sqrt{2}}{2D} > \frac{\mathrm{ln}(2)}{D}$, which is not possible according to (17). In other words, an age-synchronised steady-state population has to exhibit some variance in its interdivision time. As a consequence it can not remain age synchronised in a continuous fermenter, a result that was already conjectured by Yasuda^6^. This well-known result was also thoroughly discussed in^19,20^.

### For a self-similar batch fermenter *τ*_*obs*_ ≤ 〈*a*〉 ln(2)/(1 − ln 2)

In a closed fermenter, (2) does not have a washout term and, as a consequence, reads

$$\frac{\partial }{\partial t}n(t,l,a)+\frac{\partial }{\partial l}\,[\dot{l}n(t,l,a)]+\frac{\partial }{\partial a}n(t,l,a)+\gamma (l,a)n(t,l,a)=0$$

with the same boundary condition. Hence, *N*(*t*)’s dynamics take the form

$$\frac{dN(t)}{dt}=\iint \,\gamma (l,a)n(t,l,a)dlda$$

and *N*(*t*, *a*) follows from the same reasoning as in the previous section:

$$\frac{\partial }{\partial t}N(t,a)+\frac{\partial }{\partial a}N(t,a)+\int \,\gamma (l,a)n(t,l,a)dl=0$$

entailing *f*’s dynamics:

21 $$\begin{array}{rcl}\frac{\partial }{\partial t}f(t,a) & = & -\frac{\partial }{\partial a}f(t,a)-\frac{\int \,\gamma (l,a)n(t,l,a)dl}{N(t)}\\  &  & -\,\frac{f(a)}{N(t)}\,\iint \,\gamma (l,a)n(t,l,a)dlda\end{array}$$

For self-similar growth,

- *f* must be independent of *t*, which forces (21)’s left-hand side to vanish.
- $\iint \,\gamma (l,a)n(t,l,a)dlda$/*N*(*t*) reaches a constant value that was called *ν*_*m*_ by Powell.

Thus, for self-similar growth, (21) reads

$$\frac{df}{da}(a)=-\,{\nu }_{m}f(a)-\frac{\int \,\gamma (l,a)n(t,l,a)dl}{N(t)}$$

and the initial condition takes the form

$$f(0)=\frac{1}{N(t)}\int \,2\,\iint \,\gamma (l^{\prime} ,a^{\prime} )P(l,l^{\prime} ,a^{\prime} )n(t,l^{\prime} ,a)dl^{\prime} da=2{\nu }_{m}$$

Once again, by virtue of Duhamel’s theorem,

22 $$f(a)=2{\nu }_{m}{e}^{-{\nu }_{m}a}-\frac{1}{N(t)}{e}^{-{\nu }_{m}a}\,{\int }_{0}^{a}\,{e}^{{\nu }_{m}a^{\prime} }\,\int \,\gamma (l,a^{\prime} )n(t,l,a^{\prime} )dlda^{\prime} $$

The similarity between (22) and (16), with *ν*_*m*_ playing the same role as *D*, allows the immediate conclusion

23 $$\langle a\rangle +{\tau }_{obs}=\frac{1}{{\nu }_{m}}$$

and is accessible as soon as the cell-age and interdivision-time PDFs are measured. Furthermore, the same reasoning as in the previous paragraph yields the conclusion

24 $${\tau }_{obs}\le \frac{\mathrm{ln}(2)}{{\nu }_{m}}\iff {\tau }_{obs}\le \frac{\langle a\rangle \,\mathrm{ln}(2)}{1-\,\mathrm{ln}(2)}$$

which is the same relation between ${\tau }_{obs}$ and $\langle a\rangle$ as (19). The equality would hold if all cells were equally “healthy”. If this situation cannot be strictly ruled out, it was not observed experimentally by Powell and is highly unlikely to occur.

### Numerical examples

In this part, which deals with *E*. *coli*, all cells will be assumed cylindrical with constant diameter *d* (m) (in accordance with^6^), so that both a cell’s surface and volume are functions of *l* only. The same assumptions regarding the cell geometrical feature can also be made for *Bacillus subtilis* (as discussed in^9^). In order to put our results to the test, a comprehensive model must be formulated and simulated using either Eulerian or Lagrangian methods. We draw the reader’s attention to the fact that Lagrangian methods allow the removal of the cell age from the PBE (1) because this very feature is accessible as soon as a cell is tracked in time. In fact, Monte–Carlo methods make the model one dimension smaller and, as a result, are preferable from a computational perspective. Our Monte–Carlo simulation aims at illustrating more complex metabolic features and involves more than two variables. Notwithstanding, this has no influence on the section’s results dealing with age-related PDFs, because these extra variables can always be taken out through partial integrations. In the model used in our Monte–Carlo simulations, $\dot{{\boldsymbol{\xi }}}$ reads

$$\begin{array}{rcl}\dot{l} & = & \frac{{q}_{S}}{\rho V{Y}_{SX}}{(1-\frac{l}{\bar{l}})}^{\eta }\\ {q}_{S} & = & {q}_{{S}_{1}}+{q}_{{S}_{2}}\,{\rm{with}}\,{\rm{dynamics}}:\\ {\dot{q}}_{{S}_{1}} & = & \frac{1}{{\tau }_{1}}[{f}_{1}(S)-{q}_{{S}_{1}}]\\ {\dot{q}}_{{S}_{2}} & = & \frac{1}{{\tau }_{2}}[{f}_{2}(S){f}_{3}({q}_{{S}_{1}})-{q}_{{S}_{2}}]\end{array}$$

where *S* is the substrate concentration in the fermenter, $\rho$ a cell’s mass density (~10^3^ kg/m^3^), *V* its volume (a linear function of *l*), *Y*_*SX*_ (g/g) a (constant) substrate-to-mass ratio, and ${\tau }_{1}$, ${\tau }_{2}$ (hr) characteristic times of the respective mechanism’s adaptation. The functions *f*_1_ and *f*_2_ are of Monod shape and associate *S* to respective ${q}_{{S}_{1}}$ and ${q}_{{S}_{2}}$. The function *f*_3_ serves at a restricting factor that aims at accounting for ${q}_{{S}_{1}}$’s inhibiting influence over ${q}_{{S}_{2}}$ in accordance with^21^. This refinement aims at uncoupling the substrate uptake and lengthening at the cell scale, but is not needed for steady-state conditions. The model for $\dot{l}$ ensures that a cell divides before crossing the $l=\bar{l}$ border and the close-to-zero exponent guarantees that the lengthening phenomenon is almost linear with respect to *l* for most of the cell cycle.

Furthermore, the division frequency model is

$$\gamma (l)=\{\begin{array}{ll}\frac{1}{T}\frac{{(\bar{l}-l)}^{\kappa }-{(\bar{l}-{l}_{{\rm{\inf }}})}^{\kappa }}{{(\bar{l}-{l}_{c})}^{\kappa }-{(\bar{l}-{l}_{{\rm{\inf }}})}^{\kappa }} & {\rm{if}}\,{l}_{\inf }\le l < \bar{l}\\ 0 & {\rm{if}}\,l\notin [{l}_{{\rm{\inf }}},\bar{l}[\end{array}$$

with *T* (hr) a time constant, *l*_inf_ (m) the minimal length at rupture, and *l*_*c*_ (m) a characteristic division length.

The idea that *γ* depends only on *l* is borrowed from Robert & Al.^15^. Other assumptions have been investigated recently in the literature, such as an “adder” model^8^, which seems less convenient from a numerical simulation perspective. Indeed, due to the non-equivalent redistribution in length at rupture, such a mechanism could allow fractions of the population to grow more and more for generations on end until non-physical cell lengths are encountered.

For completeness, the redistribution kernels in *l* and *q*_*S*_ are assumed independent, beta and symmetric. To explain the first hypothesis, it is inferred from raw experimental data for two different *E*. *coli* strains^22^ that the growth rate and the length at birth are relatively independent. With little appropriated cell-scale information to the authors’ knowledge, full uncorrelation was considered, easing the analysis of the model’s sensibility to this factor. The parameters employed are given in Table 1. It can be demonstrated that the inequality −$\kappa +\eta  > 1$ entails the mathematical well-posedness of the problem. From physical grounds, this condition ensures that the rupture process overtakes lengthening as the cell length approaches the upper bound $\bar{l}$.

**Table 1:** Parameter used in the simulations.

Parameter	Value	Description	References
*D*	0.15 hr^−1^	Dilution rate	From experiment
*l*_inf_	7 × 10^−6^ m	Minimal length at rupture	^7^
*l*_*c*_	11 × 10^−6^ m	Standard length at rupture	^7^
$\bar{l}$	18 × 10^−6^ m	Maximal length at rupture	^7^
*T*	2 hr	Time scale in the cell division rate	Assumed
*Y*_*SX*_	1/0.42 ≈ 2.38 g/g	Substrate-to-mass ratio	^26^
*τ*_1_	25 s	${q}_{{S}_{1}}$ characteristic time	Assumed
*τ*_2_	5 s	${q}_{{S}_{2}}$ characteristic time	Assumed
*d*	10^−6^ m	Cell diameter	Assumed
*η*	0.05	Shape parameter	Assumed
*κ*	−0.96	Parameter	Assumed

Other elongation rate formulations, including linear or exponential laws can be found in the literature^8,9,15,23^. These laws are generally based on fitting single-cell measurements. In general none of these formulations suits the data better than the others^23^. Furthermore, Robert *et al*. evidence a sublinear elongation as the cell length approaches a critical value, what seems reasonable considering that it becomes increasingly difficult for any organism to maintain their growth rate as feeding an ever-growing cell membrane at a constant rate would likely end up mustering more resources than is available to them. Also, from a practical point of view, it is worth noticing that any experimental device introduces a bias against the older cells that are also most probably the longest.

Modelling-wise, the linear and exponential formulations imply that nothing restrains the cell elongation. In any case, the choice of the lengthening rate model must be consistent with the division frequency in order to prevent the production of cells with an infinite length. Our $\dot{l}$ and *γ* respect this constraint, even though other combinations are valid as long as the above restriction is met. In the end, however, the analytical results derived in this work do not depend on any particular choice.

With these considerations in mind, the algorithm consists in tracking the cell’s inner coordinates with respect to time, from random clipped-Gaussian initial samples. Then the division and washout events are determined by sampling two random numbers *u*, *x*:

- Let $u\sim {{\mathscr{U}}}_{[0,1]}$ sampled for each cell at each time step: mitosis occurs if $1-{e}^{-\gamma (l)\delta t} < u$
- Let $x\sim  {\mathcal E} (\tfrac{1}{D})$ sampled for each cell at birth: washout occurs should the cell’s age be greater than *x*.

When a cell divides, its inner properties are redistributed according to the kernel *K*, and each new cell is given a residence time drawn from ${\mathcal E} (\tfrac{1}{D})$. The cell age is reset to zero for one of the daughter cells, making room for a new lineage in the fermenter, whereas the other daughter keeps the record of the mother-cell’s lineage. All algorithms are coded in C++11 and the data are processed with Matlab R2016a.

### Comparison between analytical and Monte-Carlo simulations results

**Figure 2:**
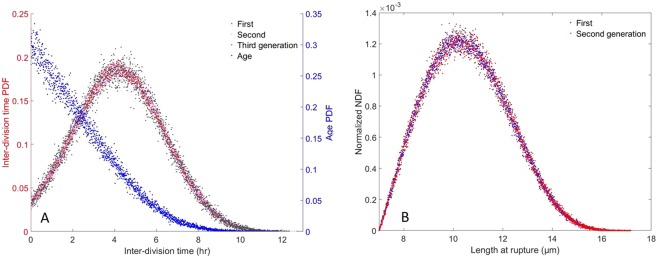
Monte–Carlo simulation results for continuous culture with $D=0.15\,{{\rm{hr}}}^{-1}$. (**A**) Steady-state cell-age (blue points) and interdivision-time *g* distributions over three generations (red, grey, light blue points) where ${\tau }_{obs}\approx 4.314\,{\rm{hr}}$. (**B**) Length distribution at division.

The Monte–Carlo simulation reaches a steady state after 4 to 5 times the slowest characteristic time ${D}^{-1}\approx 6.667\,{\rm{hr}}$. From this point onwards, consecutive division events are recorded for 1,003,306 cells over the course of 37.5 hr. Around 50% (501,322) divide at least twice and 25% (250,402) divide three times or more. This substantial database yields a numerical accuracy of approximately 10^−3^ for estimating averages. As can be seen from Fig. 1, the steady-state cell-age PDF matches many well-known results (see^12,24^ for instance), and its first moment is $\langle a\rangle \approx 2.360\,{\rm{hr}}$. In Fig. 2, the corresponding interdivision-time and length-at-division PDFs are provided, and it can be seen that both PDFs exhibit a right-skewed shape. Furthermore, the mean interdivision time can be retrieved and is approximately ${\tau }_{obs}\approx 4.314\,{\rm{hr}}$. It is worth noting that

**Figure 3:**
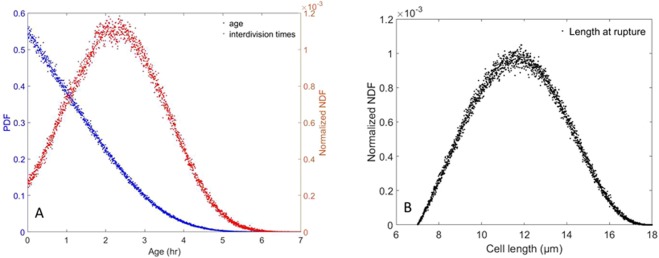
Monte–Carlo simulation results for Batch culture in the exponential-growth regime. (**A**) Cell-age PDF *f* from the Monte–Carlo simulation (blue points) and interdivision time, i.e. *g* (red points). (**B**) Length distribution at division.

- $\langle a\rangle +{\tau }_{obs}\approx 6.673\,{\rm{hr}}$. This value differs from 1/*D* by less than 0.1%.
- $$\frac{{\rm{l}}{\rm{n}}(2)}{D}\approx 4.621\,{\rm{h}}{\rm{r}} > {\tau }_{obs}$$ and $$\langle a\rangle \,\frac{{\rm{l}}{\rm{n}}(2)}{1-\,{\rm{l}}{\rm{n}}(2)}\approx 5.331\,{\rm{h}}{\rm{r}} > {\tau }_{obs}$$.
  Batch-culture simulations (cf. Fig. 3) exhibit a fairly similar pattern once exponential growth is reached. In this context, *ν*_*m*_ is retrieved from the population’s growth in mass (that is tantamount to its growth in number as mentioned in^13,25^) over a certain time interval:
  $${\nu }_{m}=\frac{\mathrm{ln}\,(\frac{m(t+{\rm{\Delta }}t)}{m(t)})}{{\rm{\Delta }}t}=\frac{\mathrm{ln}\,(\frac{N(t+{\rm{\Delta }}t)}{N(t)})}{{\rm{\Delta }}t}$$
  with *t*, *t* + Δ*t* belonging to the so-called “log phase”. In the Monte–Carlo simulation, Δ*t* = 3.28925 hr, cell mass was multiplied by 2.471 to three decimal places, and ${\nu }_{m}\approx 0.276\,{{\rm{hr}}}^{-1}$. The mean cell age and interdivision time satisfy the properties:
- $${\tau }_{obs}\approx 2.327\,{\rm{h}}{\rm{r}} < \,\frac{{\rm{l}}{\rm{n}}(2)}{{\nu }_{m}}$$
- $$\langle a\rangle \approx 1.302\,{\rm{h}}{\rm{r}} > \frac{1-\,{\rm{l}}{\rm{n}}(2)}{{\nu }_{m}}$$
- $$\langle a\rangle +{\tau }_{obs}\approx 3.629\,{\rm{hr}}$$.

In comparison, 1/${\nu }_{m}\approx 3.636\,{\rm{hr}}$, which differs from $\langle a\rangle +{\tau }_{obs}$ by less than 0.2%.

## Discussion

### Powell’s analytical results in a continuous, well-mixed fermenter

Before PBE tools were developed to address a population’s variability in different inner properties, the marginal distributions were retrieved from infinitesimal computations and Cauchy problems were extracted to be solved analytically and confronted with experimental data. Powell’s seminal article^1^ is no exception, and a relation coupling the cell-age and interdivision-time PDFs is discussed for both batch and continuous fermenters. However, (16) is not exactly the formula Powell retrieved from his own infinitesimal calculus, because the two interdivision-time PDFs do not share the same definition. Indeed, *g* is the conditional probability that, given a cell divides, it does so at age *a*, whereas Powell’s interdivision-time PDF relates to the probability that a cell divides at age *a*, the latter being less convenient in practice because it disregards the available memory from cells that reach age *a* and do not fully embrace the Markovian nature of the cell-cycle process. In the following, Powell’s *f* distribution will be labelled *h* for the sake of clarity.

A Powell-like differential equation can be devised with our definition of *g*, by starting from a set of *N* cells of which $Nf(a)da$ belong to the age interval [*a*, *a* + *da*] at time *t*. Then, during an interval of Lebesgue measure *δt*, $Nf(a)\,(1-{e}^{-D\delta t})$ cells are washed out and

25 $${\int }_{a}^{a+\delta t}\,\int \,\gamma (l)n(t,l,a^{\prime} )dlda^{\prime} ={\int }_{a}^{a+\delta t}\,g(a^{\prime} )da^{\prime} \,\iint \,\gamma (l)n(t,l,a)dlda$$

produce daughter cells of age zero (from the (3) definition of *g*). Consequently, after division by *N* on both sides,

$$\begin{array}{rcl}f(a+\delta t)-f(a) & = & -f(a)(1-{e}^{-D\delta t})-D\,{\int }_{a}^{a+\delta t}\,g(a^{\prime} )da^{\prime} \\  & = & -\delta tDf(a)-D({\int }_{a}^{\infty }\,g(a^{\prime} )da^{\prime} -{\int }_{a+\delta t}^{\infty }\,g(a^{\prime} )da^{\prime} )+o(\delta t)\\  & = & -\delta tDf(a)+\delta tD\frac{d}{da}\,{\int }_{a}^{\infty }\,g(a^{\prime} )da^{\prime} +o(\delta t)\\  & = & -\delta tDf(a)-\delta tDg(a)+o(\delta t)\end{array}$$

One immediately obtains $f^{\prime} (a)=-\,Df(a)-Dg(a)$, which mirrors (13), showing that this result is independent from the calculation methodology. It is remarkable that the reference to *g* eliminates the need to compute any conditional probability.

However, if Powell’s definition of *h*, which contains all interdivision times, is used to establish a conservation equation for the number of cells with age *a* in a reactor then one has to check that a cell has actually reached that age *a* in the system. This leads to a conditional probability and Bayes’ theorem leads to

$$\begin{array}{l}P({\rm{interdivision}}\,{\rm{time}}\le a+\delta t|{\rm{age}}\ge a)\\ \begin{array}{rcl} & = & \frac{P({\rm{interdivision}}\,{\rm{time}}\le a+\delta t\cap {\rm{interdivision}}\,{\rm{time}}\ge a)}{P({\rm{interdivision}}\,{\rm{time}}\ge a)}\\  & = & \frac{{\int }_{a}^{\infty }\,h(a^{\prime} )da^{\prime} -{\int }_{a+\delta t}^{\infty }\,h(a^{\prime} )da^{\prime} }{{\int }_{a}^{\infty }\,h(a^{\prime} )da^{\prime} }\\  & = & 1-\frac{{\int }_{a+\delta t}^{\infty }\,h(a^{\prime} )da^{\prime} }{{\int }_{a}^{\infty }\,h(a^{\prime} )da^{\prime} }\end{array}\end{array}$$

In order to reach age $a+\delta t$, any cell has to reach age *a*, remain in the system for at least *δt* and not divide between *a* and $a+\delta t$. An infinitesimal calculation using Taylor’s formula entails:

$$\begin{array}{rcl}f(a+\delta t) & = & f(a)\,\exp (\,-\,D\delta t)\tfrac{{\int }_{a+\delta t}^{\infty }\,h(a^{\prime} )da^{\prime} }{{\int }_{a}^{\infty }\,h(a^{\prime} )da^{\prime} }\\  & = & f(a)[(1-\delta tD+o(\delta t))\,(\tfrac{{\int }_{a}^{\infty }\,h(a^{\prime} )da^{\prime} +\delta t\tfrac{\partial }{\partial a}\,{\int }_{a}^{\infty }\,h(a^{\prime} )da^{\prime} +o(\delta t)}{{\int }_{a}^{\infty }\,h(a^{\prime} )da^{\prime} })]\end{array}$$

Developing and simplifying the second-order terms leads to:

26 $$\begin{array}{rcl}f(a+\delta t) & = & f(a)[1-\delta tD+\delta t\frac{-h(a)}{{\int }_{a}^{\infty }\,h(a^{\prime} )da^{\prime} }+o(\delta t)]\\  & \iff  & \frac{f(a+\delta t)-f(a)}{\delta t}\\  & = & f(a)[-D-\frac{h(a)}{{\int }_{a}^{\infty }\,h(a^{\prime} )da^{\prime} }+o(1)]\\  & = & f(a)[-D+\frac{\partial }{\partial a}\,\mathrm{ln}({\int }_{a}^{\infty }\,h(a^{\prime} )da^{\prime} )+o(1)]\\  & \mathop{\iff }\limits_{\delta t\to 0} & f^{\prime} (a)\\  & = & f(a)[-D+\frac{\partial }{\partial a}\,\mathrm{ln}({\int }_{a}^{\infty }\,h(a^{\prime} )da^{\prime} )]\end{array}$$

Straightforward computations then result in Powell’s proposed law for the relationship between the cell-age PDF and *h*, which is indeed consistent given his memoryless function *h*.

27 $$\begin{array}{rcl}f(a) & = & 2D\,\exp (\,-\,Da)\,\exp (\mathrm{ln}({\int }_{a}^{\infty }\,h(a^{\prime} )da^{\prime} )\\  & = & 2D{e}^{-Da}[1-{\int }_{0}^{a}\,h(a^{\prime} )da^{\prime} ]\end{array}$$

While all relations described in^1^ are true in both batch and continuous culture, it is of crucial importance to draw the reader’s attention to a fallacious reasoning involving Powell’s definition of *h*. The latter aims at evaluating the probability that a cell’s interdivision time is more or less than its residence time, which is determined by the relation coupling *f* and *h* in (27):

$$\begin{array}{rcl}\langle a\rangle  & = & \frac{2}{D}-2D{\int }_{0}^{\infty }\,h(a^{\prime} )\,{\int }_{a^{\prime} }^{\infty }\,a{e}^{-Da}dada^{\prime} \\  & = & \frac{2}{D}-2D\,{\int }_{0}^{\infty }\,h(a)(\frac{1}{D}a{e}^{-Da}+\frac{1}{{D}^{2}}{e}^{-Da})\,da\\  & = & \frac{2}{D}-2\,{\int }_{0}^{\infty }\,a{e}^{-Da}h(a)da-\frac{2}{D}\,{\int }_{0}^{\infty }\,{e}^{-Da}h(a)da\end{array}$$

The latter basically results in the equalities

$${\int }_{0}^{\infty }\,{e}^{-Da}h(a)da=\frac{1}{2}\,{\rm{and}}\,{\int }_{0}^{\infty }\,a{e}^{-Da}h(a)da=\frac{{\tau }_{obs}}{2}$$

This must be compared to Powell’s assumption that a cell has a probability of 1/2 of yielding two daughter cells before washout occurs, and the same probability that a cell is washed out before it begins a division event. Indeed, given that the residence time *t*_*res*_ in a well-mixed fermenter obeys an exponential law:

$${t}_{res}(t)=D{e}^{-Dt}$$

it follows that

$$P({\rm{interdivision}}\,{\rm{time}} < {\rm{residence}}\,{\rm{time}})={\int }_{0}^{\infty }\,D{e}^{-Dt}\,{\int }_{0}^{t}\,h(a)dadt$$

Then, using once again Fubini’s theorem,

$$\begin{array}{ccc}P({\rm{interdivision}}\,{\rm{time}} < {\rm{residence}}\,{\rm{time}}) & = & {\int }_{0}^{\infty }\,h(a)\,{\int }_{a}^{\infty }\,D{e}^{-Dt}dtda\\  & = & {\int }_{0}^{\infty }\,{e}^{-Da}h(a)da=\frac{1}{2}\end{array}$$

which is consistent with Powell’s result based on physical grounds.

Furthermore, the mean interdivision time is obviously not equal to ${\int }_{0}^{\infty }\,ah(a)da$ because Powell’s definition of *h* does not match the observable interdivision-time PDF. To convince oneself, the relations coupling the cell-age PDF with *g* (16) or *h* (27) entail the conclusion immediately:

$$\begin{array}{r}f(a)=2D{e}^{-Da}-D{e}^{-Da}\,{\int }_{0}^{a}\,{e}^{Da^{\prime} }g(a^{\prime} )da^{\prime} \\ f(a)=2D{e}^{-Da}-2D{e}^{-Da}\,{\int }_{0}^{a}\,h(a^{\prime} )da^{\prime} \end{array}\}\iff g(a)=2{e}^{-Da}h(a)$$

which provides the relation between *g* and *h* in a well-mixed fermenter. *g*(*a*) is conspicuously greater than *h*(*a*) if ${e}^{-Da} > 1$/$2\iff a < \,\mathrm{ln}(2)$/*D*, the reverse inequality holding if $a > \,\mathrm{ln}(2)$/*D*.

**Figure 4:**
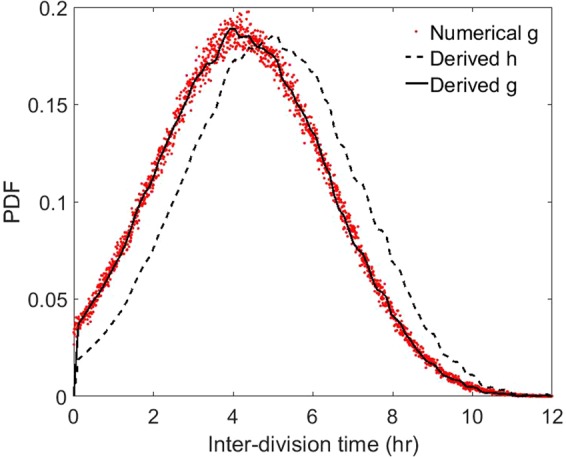
Distributions of interdivision time in Powell’s formalism: *h* (black dashed line) and its measurable counterpart *g* (black line). The numerical data retrieved from the Monte–Carlo code (red points) are shown for comparison. In general, *h* lends more weight to the older cells than *g*, so that ${\tau }_{obs} < \,\mathrm{ln}(2)$/$D < {\tau }_{uno}$.

To conclude the discussion of continuous cultures, the differences between *g* and *h* are shown in Fig. 4. Because *h* records all interdivision times, it lends weight to cells that are highly unlikely to divide in a fermenter. The observable interdivision-time PDF *g* references actual rupture events, these divisions being less and less likely as *a* approaches ln(2)/*D*. This physical reasoning testifies to the inequalities ${\tau }_{obs} < \,\mathrm{ln}(2)/D < {\int }_{0}^{\infty }\,ah(a)da$, and the relation $g(a)=2{e}^{-Da}h(a)$ allows the conclusion $g(a) > h(a)$ for $a\in [0,\,\mathrm{ln}(2)/D]$, the inverse relation being satisfied for $a > \,\mathrm{ln}(2)$/*D*.

### Painter & Marr’s inequality for the unobservable PDF

In their 1967 article^10^ addressing the interdivision-time PDF in a continuous, well-mixed fermenter, Painter & Marr incorrectly extracted the inequality ${\tau }_{obs}\ge \,\mathrm{ln}(2)$/*D* from Powell’s relation $1=2\,{\int }_{0}^{\infty }\,{e}^{-Da}h(a)da$. From their point of view, developing the exponential as a power series after factoring $2{e}^{-D{\tau }_{obs}}$ would reduce to

$$2{e}^{-D{\tau }_{obs}}\,{\int }_{0}^{\infty }\,{e}^{-D(a-{\tau }_{obs})}h(a)da=1$$

and, using the fact that the exponential function is convex,

$$2{e}^{-D{\tau }_{obs}}\,{\int }_{0}^{\infty }\,[1-D(a-{\tau }_{obs})]h(a)da\ge 1$$

Then, Painter & Marr erroneously stated that ${\int }_{0}^{\infty }\,ah(a)da={\tau }_{obs}$ to conclude. However, ${\int }_{a}^{\infty }\,ah(a)da$ is not ${\tau }_{obs}$ but refers to ${\tau }_{uno}\ge {\tau }_{obs}$ instead. Once again, the confusing definition of the interdivision-time distribution lends artificial weight to zero-measure fractions of a population.

Referring to Painter and Marr’s work, van Heerden and co-workers produced a slightly biased fit of their experimental interdivision-time PDF. Hence, their data analysis procedure involving *h* instead of *g*, lead $\langle a\rangle +{\tau }_{fit}$ to be greater than *D*^−1^ by a significant 7% margin and ${\tau }_{fit}$ to be greater than 2.259$\langle a\rangle$. However, using their raw data for *B*. *subtilis*, we find that ${\tau }_{obs}$, $\langle a\rangle$ and *D* agree with both (18) and (19). Moreover, their Supplementary Data regarding *E*. *coli* are in complete agreement with $\langle a\rangle +{\tau }_{obs}={D}^{-1}$. This analysis confirms that analytical, numerical and experimental results are in perfect agreement provided that equation (16) is used instead of (27) when dealing with a set of measured interdivision times. To conclude this discussion, it is pointed out that the experimental procedure itself affects the observed interdivision-time distribution. In Yasuda’s experiments using an optical tweezer to remove cells from the growth chamber *following their division*, no cell is washed out before dividing. Therefore, an interdivision-time distribution from such measurements resembles *h* more than the one stemming from a continuous system.

## Concluding Remarks

The exact results developed in this work throw light on the equivocal interpretations of the notion of interdivision time appearing in the literature where two different PDFs were considered from the analytical and experimental perspective. Starting from a PBE, rigorous mathematical results for the observable interdivision-time distribution have been established (complementing recent work by Jafarpour *et al*.^3^ for instance), and numerical examples are provided to supplement the theoretical results. As expected, the steady-state PDFs from the Monte–Carlo simulations proved to be in accordance with the analytical expressions. This paradigm is more suitable than Painter and Marr’s when it comes to experimental data treatment. Indeed their conclusions were based on the first moment of the unobservable cell interdivision-time distribution. The relationships provided in this work match the experimental data by van Heerden and co-workers regarding *E*. *coli* and *B*. *subtilis*.

Analysis-wise, no expression for the PDF of the cell length is accessible because the integral $\int \,\gamma (l^{\prime} )P(l,l^{\prime} )n(t,l^{\prime} )dl^{\prime}$ has no specific shape. Furthermore, with two relations pertaining to *P*:

1. $$P(l,l^{\prime} )=P(l^{\prime} -l,l^{\prime} )$$
2. $${\int }_{0}^{l^{\prime} }\,P(l,l^{\prime} )dl=1$$

one can extract the dynamics of the length distribution’s zeroth and first-order moments only, with the help of integrations by parts and Fubini’s theorem. However, no additional formulae are available if no other relations constrain *P*.
